# Leveraging hybrid biomarkers in clinical endpoint prediction

**DOI:** 10.1186/s12911-020-01262-3

**Published:** 2020-10-07

**Authors:** Maliazurina Saad, Ik Hyun Lee

**Affiliations:** 1 University of Illinois at Urbana-Champaign, 1406 W. Green St, Urbana, IL 61801 USA; 2 Korea Polytechnic University, 237 Sangidaehak-ro, Siheung-si, Gyeonggi-do 15073 South Korea

**Keywords:** Predictive models, Clinical decision- making, Biomarker, Imaging, Endpoint

## Abstract

**Background:**

Clinical endpoint prediction remains challenging for health providers. Although predictors such as age, gender, and disease staging are of considerable predictive value, the accuracy often ranges between 60 and 80%. An accurate prognosis assessment is required for making effective clinical decisions.

**Methods:**

We proposed an extended prognostic model based on clinical covariates with adjustment for additional variables that were radio-graphically induced, termed imaging biomarkers. Eight imaging biomarkers were introduced and investigated in a cohort of 68 non-small cell lung cancer subjects with tumor internal characteristic. The subjects comprised of 40 males and 28 females with mean age at 68.7 years. The imaging biomarkers used to quantify the solid component and non-solid component of a tumor. The extended model comprises of additional frameworks that correlate these markers to the survival ends through uni- and multi-variable analysis to determine the most informative predictors, before combining them with existing clinical predictors. Performance was compared between traditional and extended approaches using Receiver Operating Characteristic (ROC) curves, Area under the ROC curves (AUC), Kaplan-Meier (KM) curves, Cox Proportional Hazard, and log-rank tests (*p*-value).

**Results:**

The proposed hybrid model exhibited an impressive boosting pattern over the traditional approach of prognostic modelling in the survival prediction (AUC ranging from 77 to 97%). Four developed imaging markers were found to be significant in distinguishing between subjects having more and less dense components: (*P* = 0.002–0.006). The correlation to survival analysis revealed that patients with denser composition of tumor (solid dominant) lived 1.6–2.2 years longer (mean survival) and 0.5–2.0 years longer (median survival), than those with less dense composition (non-solid dominant).

**Conclusion:**

The present study provides crucial evidence that there is an added value for incorporating additional image-based predictors while predicting clinical endpoints. Though the hypotheses were confirmed in a customized case study, we believe the proposed model is easily adapted to various clinical cases, such as predictions of complications, treatment response, and disease evolution.

## Background

The prediction of clinical endpoints or outcome measures has always been the focus of personalized medicine, as well as the key learning applications of ill-health related studies, in an effort to provide clinicians with simple and reproducible risk assessment models. It plays important roles in the clinical decision support system, as it is closely related to the interventions or therapeutic selection, care-planning, and resource allocation [1, 2]. An outcome measure as defined in clinical practice is any characteristic or quality measured as the result of health interventions to assess the impact on a patient’s health status [3], such as the survival period, recurrence or relapse of a cancer, or adverse events. Previous attempts to predict a patient outcome mostly centered to a mortality risk stratification (probability of death), especially for severely ill patients that are admitted to an intensive care unit (ICU) [4–7]. There exist prognostic instruments available such as palliative prognostic score (PaP) [8], palliative prognostic index (PPI) [9], acute physiology and chronic health evaluation (APACHE), [10] and simplified acute physiology score (SAPS) [11]. However, these tools have significant shortcomings because they are derived from a population of patients that are already determined to be terminally ill, making them less relevant for patients who are still receiving anti-cancer treatment [12].

Generally, an outcome prediction model is developed using one of these two approaches: I) patient similarity or II) predictive modeling. A patient similarity-based model makes predictions by identifying and analyzing past patients who are similar to a present case through a correlation metric [13, 14]. On the other hand, a predictive modeling requires the extraction of features of interest, followed by the modeling of desired outcome using machine learning algorithms [15, 16]. Several studies have demonstrated the comparison of patient similarity vs. predictive modeling, for which the latter outperformed the former in terms of predictive values [17, 18]. Traditionally, clinical prognosis has been derived from clinical covariates or biomarkers available in the electronic medical record (EMR), that usually cover a variety of aspect of a patient’s health state such as vital signs, physiological variables, demographic information, and laboratory test results [12, 13, 17, 18]. These approaches resulted in accuracy between 60 and 80% [19, 20]. Nonetheless, radiographically induced biomarkers have started to show potential in prognostic models [21–23]. The latter claimed to be at advantage due to its non-invasive nature while showing improvement over the traditional approach. In addition, EMR often contains many missing values, imposing great challenges in the traditional method.

In the present study, we seek to investigate the prognostic impact provided by clinical biomarkers (CBMs) and imaging biomarkers (IBMs), as well as the hybrid of both, termed hybrid biomarkers (HBMs). Our hypotheses are as follows: (a) IBMs deliver better discrimination power in a clinical prognostication in comparison to CBMs, (b) IBM approach of modelling the prognostic model is general and applicable to different kind of patient outcome prediction, though our main focus in this work is the survival prediction, and (c) there is an added value provided by IBMs in combination with CBMs while making prediction of patient outcomes. The group of patients targeted to validate these hypotheses are taken from a public Non-Small Cell Lung Cancer (NSCLC) archive. Post-surgical prognostic models are developed for patients who show signs of nodule internal features such as cavitation, cysts, reticulation and air bronchogram pre-surgery. The motivation behind this targeted group is to investigate if the pre-operative radiographic features can predict tumor invasiveness based on air/gas to tissue proportion and thus, aiding physicians to determine the most appropriate surgical procedure in such cases. As the literature indicates, lung cancers that show wider section of radiolucency such as Ground Glass Opacity (GGO) are considered to have more favorable diagnosis than solid tumors [24, 25]. The objective of this study is however not to re-iterate what is already known in the literature, but to explore the possibility of improving clinical decisions through an enhanced prognostic model using a collection of so-called informative image-based covariates as well as establishing their relationship to clinical endpoints.

## Methods

### Clinical materials

Imaging and clinical records of patients diagnosed with primary NSCLC, who received surgical excision were obtained from a public repository, the cancer imaging archive (TCIA) [26]. The cohort consists of 211 subjects that underwent both computed tomography (CT) and Positron Emission Tomography/Computed Tomography (PET/CT) scans. Semantic annotations and segmentation maps of the tumor were available. Inclusion criteria encompassed subjects of stage I-IV cancer with either cysts, cavitation, reticulation, or air bronchogram sign. There were 48 males and 20 females with mean age of 69.5 and 66.5 years, respectively. Subjects without tumor internal characteristics or incomplete records were all excluded. Five-year survival was calculated from the day of surgery to the last follow-up date. The dataset’s characteristics are supplemented in Table 1. The acquisition protocol varied slightly for different patients, depending on each patient’s size. Exposure settings were constant at 120 kVp, with tube current ranging from 50 to 750 mAs. Pixel spacing and slice thickness ranged from 0.596 to 0.976 mm and 0.625 to 3.75 mm respectively. The images were reconstructed at 512 × 512-pixel matrices.

**Table 1:** The details of dataset used in this work

Dataset characteristics	No	(%)
**Total patients**	211	100
Incomplete records	50	24
Complete records	161	76
**Internal features present**	68	42
I) Air Bronchogram	24	35
II) Cavitation	15	22
III) Cysts	4	6
IV) Reticulation	5	7
V) Mix of above	20	29
**Internal features absent**	91	58
**Gender**		
Male	(48/68)	71
Female	(20/68)	29
**Age**		
≤ 70	(36/68)	53
> 70	(32/68)	47
**Pathological staging**		
Primary tumor T		
I	(30/68)	44
II	(25/68)	37
III	(9/68)	13
IV	(4/68)	6
Lymph node N		
0	(56/68)	82
1	(4/68)	6
2	(8/68)	2
3	0	0
Metastasis M		
0	(66/68)	97
1	(2/68)	3
**Histology**		
Squamous cell	(10/68)	15
Non-Squamous cell	(58/68)	85
**5-year overall survival**		
Survived	(23/68)	34
Expired	(45/68)	66

### Tumor delineation

**Fig. 1:**
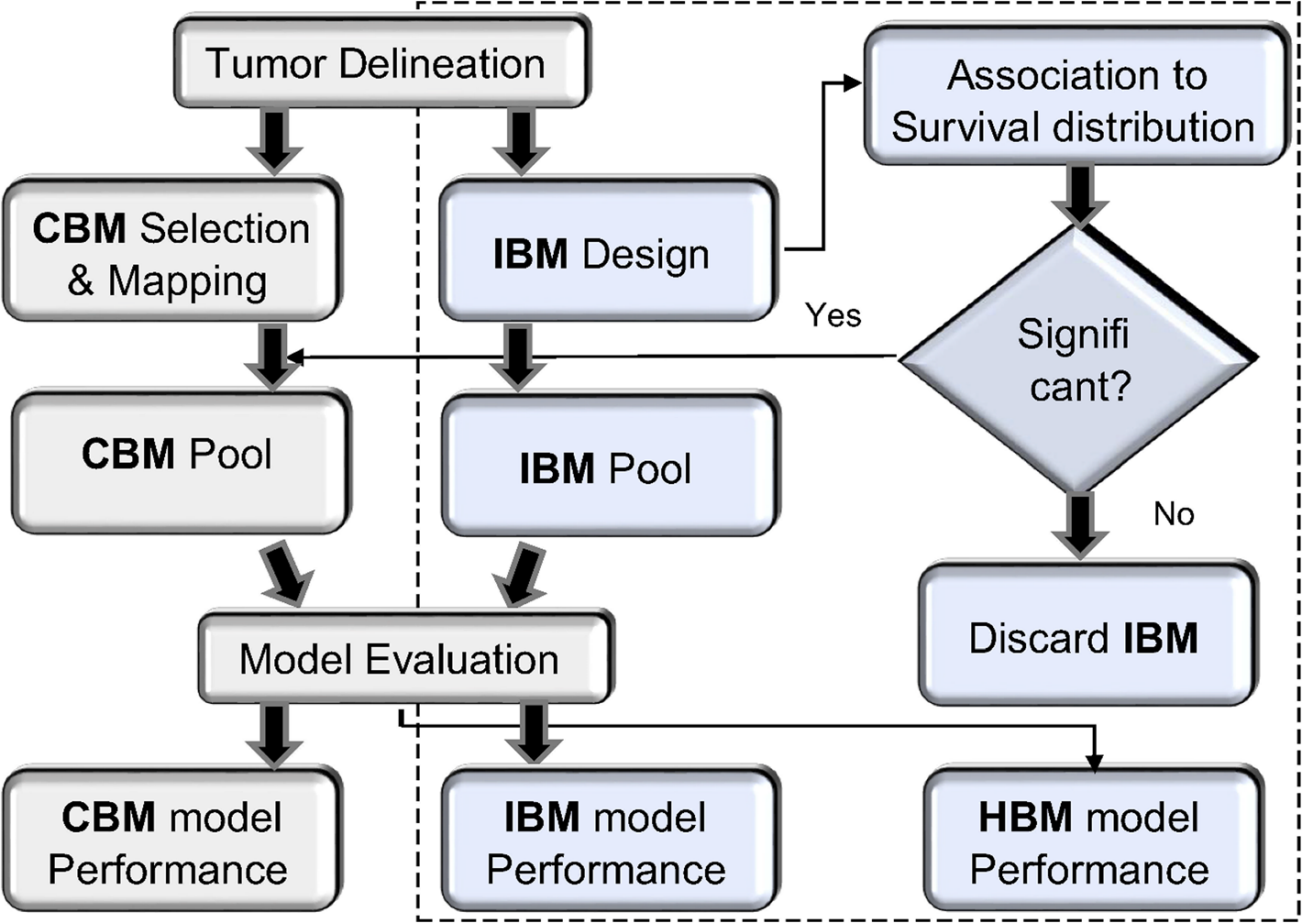
Flow diagram of the proposed model. The extended framework is indicated on the right side of the model, inside the dotted lines

Figure 1 outlines the processes required to achieve the objectives of this study. Tumor delineation was a pre-processing step and was performed using an automated tool developed using geometrical and topological processing to facilitate this process [27]. It eliminates the manual delineation work required in this study.

### CBM selection and mapping

Two main models were referenced during the selection of clinical biomarkers in this work: Wallington et al. [19] and Jochems et al. [20]. Both investigated the prediction of survival in NSCLC patients using CBM readily available from a patient’s record such as demographics, tumor staging, tumor size, and treatment history. Wallington’s model used age, gender, BMI, tumor staging, income deprivation, performance status, and history of previous treatment as the predictors, whereas Jochems’s model used age, gender, tumor size, tumor staging, total dose, performance status, and chemo-timing. Following their guidelines, we have chosen the nine CBMs most similar to their studies that were available in our dataset, as depicted in Table 1. Accordingly, some of these variables had to go through a feature transformation in order to map those that mostly exist either in nominal or categorical form to more classifier-friendly variables. Gender, for instance, went through a transformation from nominal (female, male) to categorical (0, 1) values while TNM-staging went through a mapping from categorical values (1,2,3,4) to numeric values calculated based on the percentage of their composition in the dataset. This is performed to avoid matrix sparsity. The details of each clinical variable mapping work are presented in Table 2.

**Table 2:** CBMs selection and their mapping work

Covariates	Type	Range	Conversion
Gender	Nominal	{Male, Female}	(0,1)
Age	Real	(42–87)	NA*
Weights (lbs)	Real	(80–318)	NA
Smoking years	Real	(0–41)	NA
Histology	Nominal	{Squamous, Non-Squamous}	(0,1)
T	Categorical	[1–4]	Number of patients in a stage divided by total number of patients (e.g., 16 patients categorized as T1; those patients were given 0.235 (16/68) value).
N	Categorical	[0,1,2,3]	
M	Categorical	[0,1]	
Tumor size (mm)	Real	(11.7–73.9)	NA

*NA means no conversion work is needed

### IBMs design

**Fig. 2:**
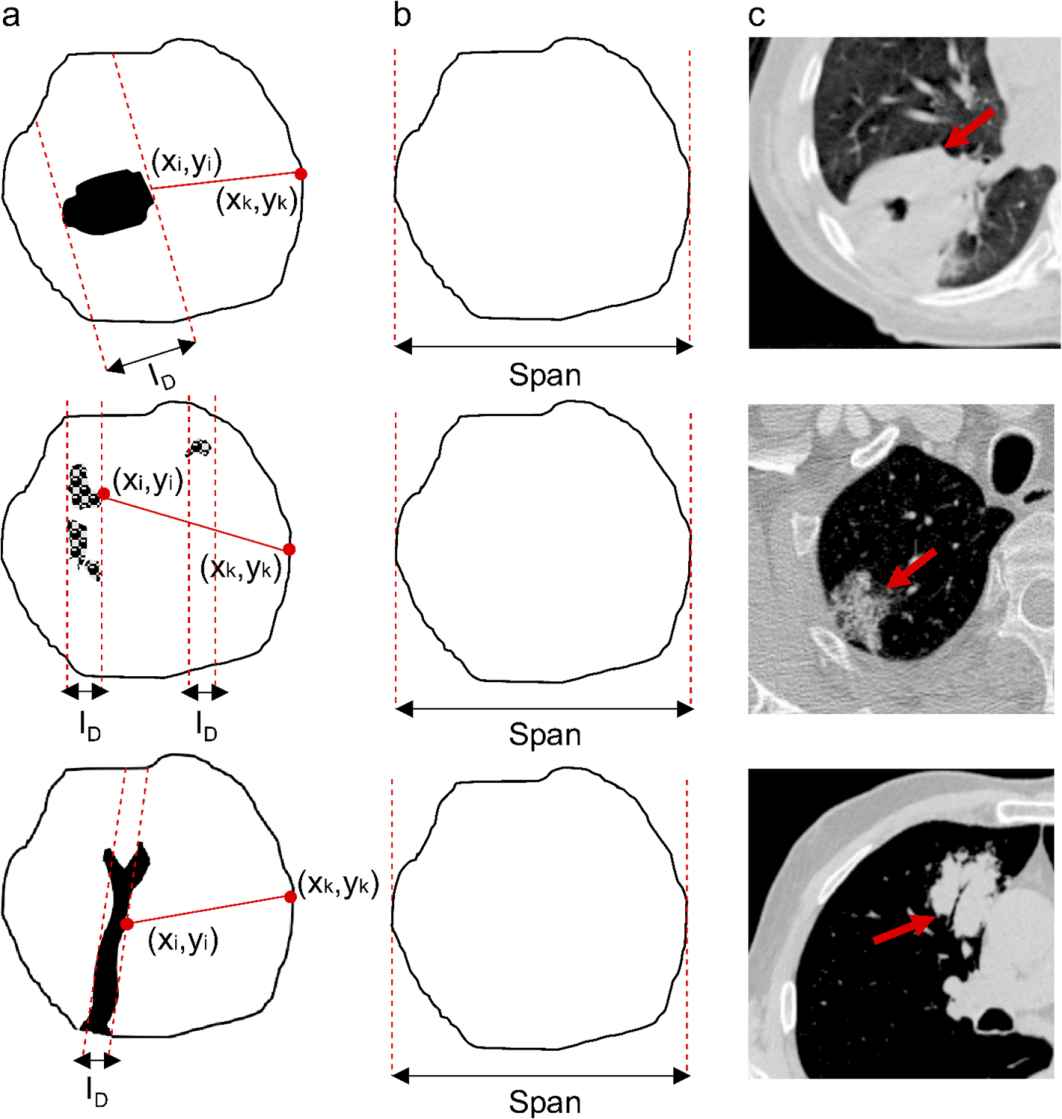
Example of decreased density areas in a tumor. Rows from top to bottom represent cavitation, reticulation, and air-bronchogram sign phenomena, respectively. Meanwhile columns A to C show illustration of the internal features, the tumor mask, and an example of CT scan for each case. The solid and non-solid components are designated by white and black colors in the first column, respectively

The areas of decreased density in computed tomography are described by specific radiologic lexicons such as cavitation, cysts, reticulation, and air bronchogram signs. Many of these terms are based on the pathogenesis and the opacification characteristics possessed by the lung abnormalities. The decreased in a nodular attenuation pattern develops when the density in parenchyma decrease caused by: (a) abnormal increase in the amount of air, (b) abnormal decrease in blood volume, or (3) loss of soft tissue structures. Each of these phenomena may results in different pattern of decreased density for instance those depicted in Fig. 2 (c).

To validate our hypothesis, eight customized IBMs were introduced in this work to quantify the solid and non-solid composition of a tumor with internal characteristic. The internal features included were cysts (radiolucency with a thin wall), cavitation (radiolucency with a thick wall), reticulation (lucent spaces created by the intersection of fine, medium or coarse lines) and air-bronchogram sign (gas-filled bronchi surrounded by alveoli filed with fluid, pus or other substances). The gray-level images are first converted to binary images as depicted by a few examples in Fig. 2.a. We created a tumor mask for each binary tumor as depicted by Fig. 2.b. Table 3 shows the definition of covariates we used to design the imaging biomarkers, where we used the term *A* and *B* to denote binary images (Fig. 2.a) and mask images (Fig. 2.b), respectively. Our fundamental approach is simple in which the white pixels in both *A* and *B* refer to solid areas of tumors, whereas the black pixels refer to the non-solid areas. *PA* and *PB* represent the counts of pixel of *A* and *B*.

**Table 3:** The list of covariates used to derive eq. 1, 2, 3, 4, 5, 6, 7 and 8. Active pixels refer to the white pixels in binary images

Covariates	Definition
P_A_	The number of active pixels in A
P_B_	The number of active pixels in B
P (_B∩A_)	The number of active pixels that are true for both A and B
n	The number of effected slices
(x,y)	The contour vertices: - - Air pocket contour vertices (xi,yi) - Solid wall contour vertices (xk,yk)
Span	The longest distance between two vertices of the tumor mask
I_D_	Inner diameter of a lucent area. If more than one area is present, the average is calculated.

Equation 1 measures the proportion of solid component (radiodensity) of a tumor with internal features. The solid components are those that appear opaque white or grey in CT scans. RDC should range between 0 and 1.

1 $$ RadioDense\ Composition\ (RDC)=\frac{\sum_{i=1}^n\left({P}_A/{P}_B\right)}{n} $$

A and B are depicted in Fig. 2.

Equation 2 is an IBM that quantifies the proportion of decreased density areas of a tumor with internal features. These so-called non-solid components appear as black in CT scans. Similarly, RLCs fall between 0 and 1 ranges. *RDC*, and *RLC* are inter-correlated in a way that each calculates the ratio of radio-density and radio-lucency of the same tumor area.

2 $$ RadioLucent\ Composition\ (RLC)=\frac{\sum_{i=1}^n\left({P}_B-{P}_A\right)/\left({P}_B\right)}{n} $$

Difference in composition as depicted in Eq. 3 calculates the difference in the solid and non-solid composition in Eq. 1 and 2.

3 $$ Difference\ of\ Composition(DoC)=\sqrt{\Big( RDC-{RLC}^{\Big)2}} $$

An IBM computing the fraction of radiolucency to radiodensity (non-solid to solid) areas is introduced in Eq. 4. It measures the air to tissue ratio of a tumor.

4 $$ Air\  to\ Tissue\ ratio\ \left({AT}_R\right)=\frac{\sum_{i=1}^n\left({P}_B-{P}_A\right)/P\left({}_{B\cap A}\right)}{n} $$

Length of the solid area as shown in Eq. 5 searches for the longest path between the radio-lucent to the radio-dense boundaries. It refers to the wall of a tumor with internal features. If more than one radio-lucent area is present, the algorithm chooses the largest one to computes the boundary vertices.

5 $$ Length\ ofSolid\ Area(LoSA)=\mathit{\operatorname{Max}}\left({\sum}_{i=1}^{\mathrm{n}}\sqrt{{\left( xi- xk\right)}^2+{\left( yi- yk\right)}^2}\right) $$

The sixth biomarker as depicts in Eq. 6 quantifies the ratio of *LoSA* to the diameter of a tumor mask (Span), where no lucent area is observed.

6 $$ Length\ of\ of Solid\ Area\ ratio\ \left({LoSA}_R\right)=\frac{\mathit{\operatorname{Max}}\left({\sum}_{i=1}^n\sqrt{{\left(x\mathrm{i}- xk\right)}^2+{\left( yi- yk\right)}^2}\right)}{Span(B)} $$

To gain further insight of the possible differences between solid vs non-solid components of a tumor, IBMs measuring the length of a lucent area as well as the ratio of their averaging length (for multi-lucency cases) to the diameter of the tumor were also investigated. Eq. 7 demonstrates the average length of cavities, whereas Eq. 8 shows the quantification of the averaged diameter of the lucent areas over the diameter of the tumor mask (B in Fig. 2)

7 $$ Length\ of\ C\mathrm{a} vity\ Area\ (LoCA)=\frac{\sum_{i=1}^n AVG\left({I}_D\right)}{n} $$

8 $$ Length\ of\ Cavity\ Area\ ratio\ \left({LoCA}_R\right)=\frac{\Big({\sum}_{i=1}^n AVG\left({I}_D\right)/n}{Span(B)} $$

In order to match the number of CBMs, we included an existing measurement in literature, solidity as the final IBM. Solidity calculates the ratio of true pixels between the tumor and its bounding box.

### Model evaluation

To test the first hypotheses, three set of test cases were drawn as shown in Table 4. The predictors were fed into four off-the-shelf classifiers to predict the probability of patients *survived* or *expired* 5 years after surgery. The classifiers were chosen based on recent similar published works of predicting survival of lung cancer: Wallington et al., Logistic Regression (LR) [19], Jochems et al., Random Forest (RF) [20], Hazra et al., Support Vector Machines (SVM) [28], and Rodrigo et al., Artificial Neural Network (ANN) [29].

**Table 4:** The set of predictors forming three test cases; CBM, IBM, and HBM. The HBM are a combination of CBMs and selected IBMs

CBM pool	IBM pool	HBM pool		
Age	RDC	Age	+	Selected imaging biomarkers based on correlation testing.
Gender	RLC	Gender		
Weights	DoC	Weights		
Smoking years	AT_R_	Smoking years		
Histology	LoSA	Histology		
T stage	LoSA_R_	T stage		
N stage	LoCA	N stage		
M stage	LoCA_R_	M stage		
Tumor size	Solidity	Tumor size		

The predictive performance was evaluated using a cost-sensitive measure which is area under the Receiver Operating Characteristic (AUROC) or AUC. Cost insensitive measures such as accuracy, precision, and recall, might be biased in our case due to the nature of the dataset that is skewed towards one class. AUC is visualization tool for which may appropriately determine the appropriateness of a classifier. On top of that, to mitigate the concern on skewed dataset, the 10-fold cross validation was incorporated to stratify the samples and ensure that the ratio between positive and negative case in each fold are similar to that in the entire dataset. In other words, the dataset is first divided into two strata, then random assignment to the folds is carried out in each stratum independently [30]. Integrated discrimination improvement (IDI) was implemented to measure the significant difference, if it exists, between the AUCs returned by each model [31]. IDI index is commonly used to compare two risk prediction models or taking the difference between two competing models. For instance, when comparing the CBM-based model to the IBM-based model performance, the index tells the improvement in prediction without the inherit problems of directly comparing c-statistics.

### Correlating IBMs to survival distribution

In this section, the prognostic impact of the proposed IBMs performed through uni- and multi-variable analyses, with the chi-squared test or D’Agosstino-Pearson opted for normality testing for categorical and continuous variables, respectively. The Kaplan–Meier (KM) survival curve, Cox Proportional Hazard model, and the log-rank test were the methods used to investigate these correlations. KM estimator used to estimate the survival function from life-time data, for example, the fraction of patients living for a certain amount of time after treatment [32], while the log-rank test is a hypothesis test to compare the survival distribution between two groups. Both KM and the log-rank test are examples of univariable analysis and non-parametric statistic. They describe the survival according to one factor under investigation but ignore the impact of another. Cox Proportional Hazards is a regression model that extends survival analysis to assess simultaneously, the effect of several factors on survival time. It allows the examination on how specific covariate influence the event of interest at a particular point of time. The rate is known as hazard ratio [33]. Descriptive data were summarized as mean and median with 95% confidence interval, while categorical data was given as count or proportion. Statistical significance is given by a two-tailed *p*-value lower than 0.05.

**Fig. 3:**
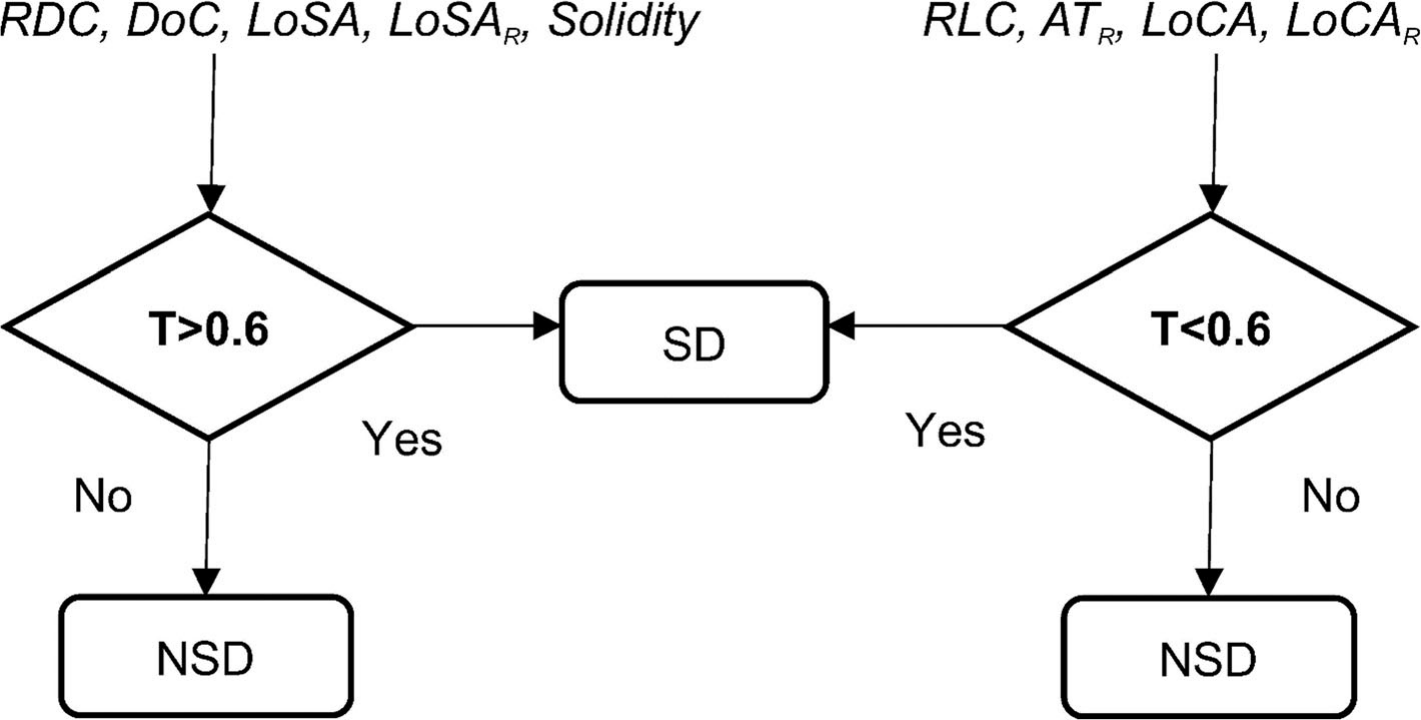
Two sub-groups created based on the threshold by each IBM. In the case a measurement that is not a ratio, such as LoSA and LoCA, a posterior probability is calculated prior the threshold setting

The subjects were divided into two comparative groups using the proposed IBMs. *RDC*, *DoC*, *LoSA, LoSAR*, and *Solidity* are IBMs that are associated to quantify denser composition of a tumor, whereas *RLC*, *ATR*, *LoCA*, and *LoCAR*, are the IBMs that concentrated on quantifying the less dense portion of a tumor. All IBMs measurements are in the range of 0–1 hence a threshold of 60% (0.6) was set to divide the subjects into two groups that we named as solid dominant (SD) and non-solid dominant (NSD). Figure 3 presents how this work took place. All algorithms were implemented using MATLAB (R2015b) and all statistical analyses were conducted using MedCalc software version 17.5.5.

## Results

### Prediction of 5-year survival

The prediction of 5-year survival were evaluated between models that were built based on CBM versus models built using the proposed IBM. Table 5 depicts the performance metric AUC and IDI for the prediction of post-surgery survival for patients with nodule internal features by both methods. All four classifiers demonstrated significant improvement in the proposed IBM model with IDI ranged between 0.47–0.54 (*p* < 0.05) in which the ANN and RF showing the highest jump from CBM to IBM model. IBM seemed to successfully boost the prediction accuracy above 0.80 in all classifiers tested in comparison to its counterpart, CBM model that demonstrated AUC ranged between 0.59–0.75. We observed that Logistic Regression outperformed the Random Forest classifier in both CBM and IBM models, which is actually contradictory to the finding in Jochems et al. [20] that we used as our main reference. Interestingly, in terms of the percentage of improvement, Random Forest is indeed among the classifiers having significant boosting performance (*p* < 0.001). We believe parameters tuning has something to do with these observations, as Random Forest required a few parameters to be tuned, for instance, the number of branches. This experiment confirmed our first hypothesis.

**Table 5:** Performance comparisons of AUC for the survival prediction in both models

Classifiers	CBM (AUC)	IBM (AUC)	IDI	***P***-value
Logistic Regression	0.75	**0.93**	0.47	0.002*
Random Forest	0.61	**0.83**	0.54	< 0.001*
Support Vector Machine	0.74	**0.92**	0.50	< 0.001*
Artificial Neural Network	0.59	**0.82**	0.52	< 0.001*

* representing significance data

### Association to survival ends

We have seen IBM outperformed CBM method in survival prediction. To derive further insights on the probable reason underlying this observation, association test was conducted on both type of biomarkers. Tables 6 and 7 depict the correlation of CBM and IBM to overall survival respectively. The univariable analysis has demonstrated that four CBMs were found to be significant factors predicting the overall survival in the studied case; age [HR: 2.235, (r = 0.26, *p* = 0.037)], lymph node involvement [HR: 3.797, (r = 0.23, *p* = 0.056)], metastasis event [HR: 4.863, (r = 0.11, *p* = 0.368)], and tumor size [HR: 1.059, (r = 0.37, *p* = 0.002)]. The multivariable analysis only retained age and lymph node involvement from this pool; [χ^2^ (2) =14.498, *p* = 0.0007]. With these observations, we have concluded that age and lymph node involvement were the risk factors useful in the survival prediction.

**Table 6:** Uni- and multi-variable survival analysis for CBMs were performed through KM and Cox proportional hazard model, respectively

Biomarkers	Univariable		Multivariable	
	HR	95% CI	HR	95% CI
**Demographic factors**				
Age				
≤ 70	1		1	
> 70	2.235	1.097–4.556^a^	2.257	1.240–4.111
Gender				
Male	1			
Female	1.015	0.560–1.837		
Weights				
≤ 150	1			
> 150	1.070	0.604–1.897		
Smoking Status				
Yes	1			
No	1.623	0.872–3.021		
**Clinical factors**				
Primary Tumor				
≤ T2	1			
> T2	0.904	0.446–1.830		
Lymph Node				
N0	1		1	
≥ N1	3.797	1.038–13.887^a^	4.163	1.858–9.326
Metastasis				
M0	1			
M1	4.863	0.238–99.479^a^		
Histology				
Squamous	1			
Non-Squamous	1.194	0.582–2.452		
**Tumor size**				
Longest diameter	1.059	1.010–1.110^a^		

* representing significant data; *HR* Hazard ration, *CI* Confidence interval

**Table 7:** Uni- and multi-variable survival analysis for IBMs were performed through KM and Cox proportional hazard model, respectively

Biomarkers	Univariable		Multivariable	
	HR	95% CI	HR	95% CI
**Solid Composition**				
RDC				
SD	1		1	
NSD	2.225	1.149–4.307*	0.431	0.232–0.769
DoC				
SD	1			
NSD	1.583	0.749–3.342		
LoSA				
SD	1			
NSD	1.963	1.019–3.781		
LoSA_R_				
SD	1		1	
NSD	2.445	1.345–4.443*	0.395	0216–0.708
Solidity				
SD	1			
NSD	1.908	0.930–3.915*		
**Lucent Composition**				
RLC				
SD	1			
NSD	2.225	1.149–4.307*		
AT_R_				
SD	1			
NSD	2.018	1.011–4.028*		
LoCA				
SD	1			
NSD	0.643	0.337–1.225		
LoCA_R_				
SD	1		1	
NSD	2.274	1.153–4.488*	0.422	0.232–0.769

* representing significant data; *HR* Hazard ration, *CI* Confidence interval

On the other hand, only *DoC*, *LoSA*, and *LoCA* were not statistically significant in predicting survival between two groups (SD vs. NSD) using IBMs. We also observed that two IBMs; *RDC* and *RLC* showing similar prognostic impact [HR: 2.225, (r = ±0.67, p < 0.0001)], which was believed due to the reason that they are correlated to each other in a way that one complements another. Multivariable analysis retained three IBMs from the significant pool which were *RDC*, *LoSA*_*R*_, and LoCA_R;_ [χ^2^ (3) =42.631, *p* < 0.0001]. Following these observations, the comparison of mean and median survival time between the subjects grouped were also investigated and shown in Table 8. It was observed that the groups differed between 1.64–2.23 years in the mean survival and 0.46–2.00 years in the median survival. The survival curves are supplemented in supplementary file S1.

**Table 8:** Mean and median survival as calculated from Kaplan-Meier survival curves. Only IBM which gives statistical significance as demonstrated by the univariate analysis in Table 7 is included in this table

Biomarkers	5-Year Overall Survival					
	Mean	95% CI	Difference	Median	95% CI	Difference
**Solid Composition**						
*RDC*						
SD	5.20	4.27–2.57	2.04	4.43	2.93–5.48	1.17
NSD	3.16	2.57–3.74		3.26	2.49–3.90	
LoFA_R_						
SD	5.50	4.46–6.61	2.23	5.20	3.11–5.24	2.00
NSD	3.27	2.75–3.79		3.20	2.71–3.84	
*Solidity*						
SD	4.96	4.08–5.85	1.64	3.90	2.85–5.48	0.58
NSD	3.32	2.68–3.96		3.32	3.15–3.84	
**Lucent Composition**						
*RLC*						
SD	5.20	4.27–2.57	2.04	4.43	2.93–5.48	1.17
NSD	3.16	2.57–3.74		3.26	2.49–3.90	
*AT*_*R*_						
SD	5.05	4.11–5.99	1.69	3.78	2.76–5.48	0.46
NSD	3.36	2.82–3.90		3.32	3.15–3.90	
*LoCA*_*R*_						
SD	5.17	4.24–6.11	2.03	3.99	3.32–5.48	0.83
NSD	3.14	2.59–3.69		3.16	2.49–3.84	

Data are presented in years

### Leveraging the hybrid biomarkers for patient outcome predictions

**Fig. 4:**
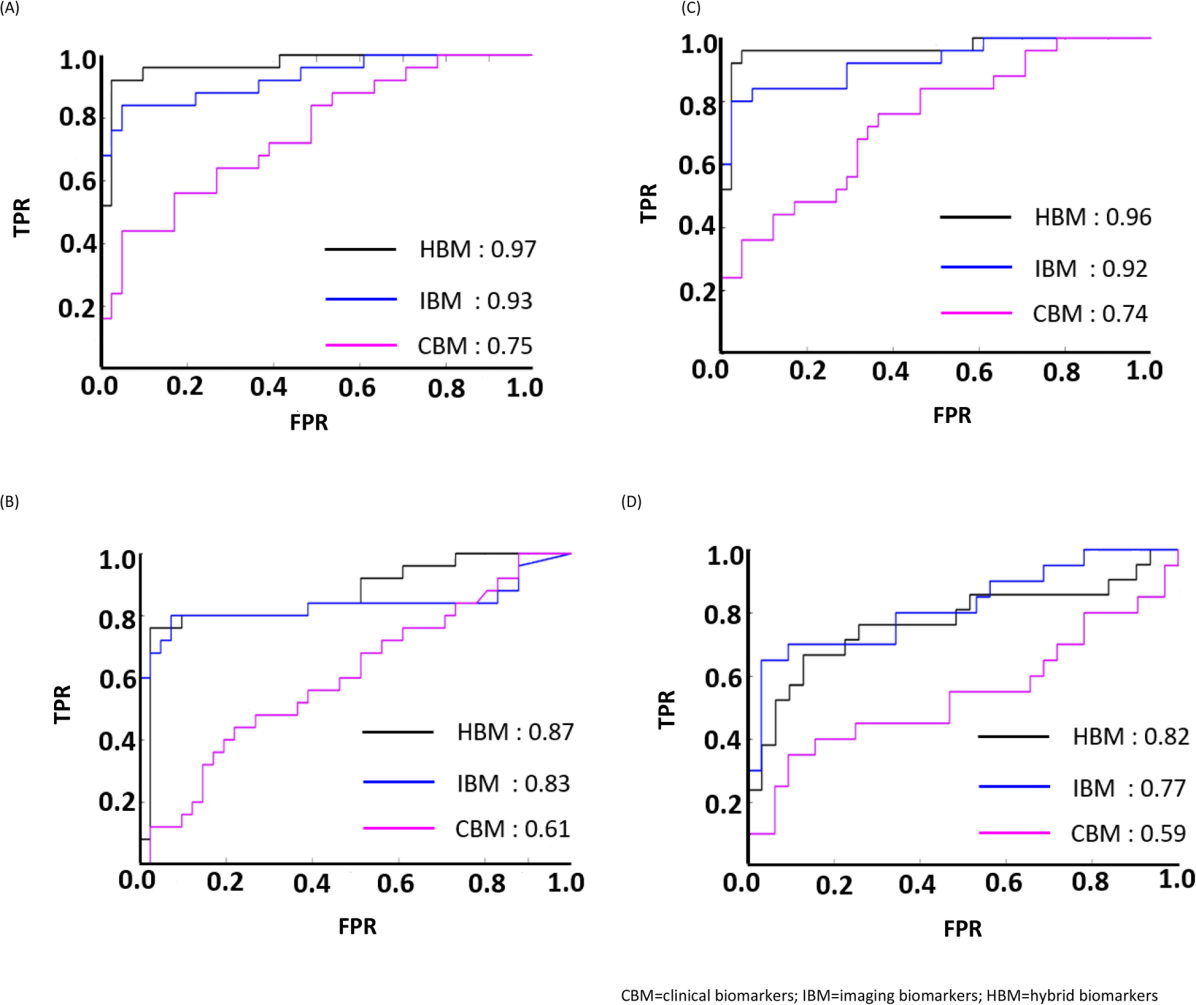
The comparison of ROC curves in the survival prediction for all models in: **a** Logistic Regression, **b** Random Forest, **c** Support Vector Machines and **d** Artificial Neural Network

Based on findings in the previous section, four IBMs (*RDC*, *RLC*, *LoSA*_*R*_, *LoCA*_*R*_) with prognostic values were leveraged into a hybrid model that combine them with all of the clinical predictors. This hybrid model is termed as hybrid biomarkers (HBM). Similar survival predictions were conducted and the ROC curves and AUCs were plotted to compare the performance of all three models. Figure 4 demonstrates the comparison between all models for the survival prediction. We observed that HBM based model, to some extent boosted the performance of IBM further by 4% in all but ANN classifier. Though HBM managed to surpass the performance of CBM based model, IBM was seen to work best for ANN.

## Discussion

We have investigated the efficacy of combining archival clinical data with radiographically induced data in personalizing the risk stratification of NSCLC patients undergoing anti-cancer treatment. Eight image-based biomarkers were introduced customized to the case being studied, in which six demonstrated statistical significance in the mean and median survival. This finding could be a useful input for the precision medicine community in identifying patients with higher risk to be put under additional therapeutic planning. The proposed biomarkers may provide alternative factors for oncologists investigating tumor-specific factors during treatment planning, which is a less-invasive method than biopsy or resection sampling.

The results support all hypotheses made in which the imaging measures are superior predictors in comparison to clinical measures, and thus confirms the utility of incorporating image-based predictors into the traditional approach of using clinical-based predictors in modeling the patient outcome prediction. Although significant improvement was observed in the image-based predictive model over the traditional model, the hybrid between them was seen to outperform both standalone models, in most cases. Although there was a standout case in ANN classifier, where the hybrid predictors fall slightly short behind imaging predictors, which merits further investigation, this does not forfeit the third hypothesis since the clinical predictors still underperformed in comparison to the hybrid predictors.

Demographics, histology and pathological staging are among clinical indicators that have been proven in the literature [34–36], hence previous works on predicting survival among NSCLC patients are concentrated on mixing these readily available clinical factors with AUCs range between 0.62–0.79 [19, 20, 27, 28]. To the best of our knowledge, this is the first study establishing the fusion of both clinical with imaging covariates, which has been proven to better predict survival (AUCs between 0.77 and 0.97). Even though the concept of imaging measures as biomarkers is not relatively new [22, 23, 37], the thought of having to go through complex imaging analysis with advanced software might have hindered the unique benefit it may provide. Imaging biomarkers are the corner stone of modern radiology. They might be a major player in therapeutic decisions and drugs evaluation in the near future; thus, multi-disciplinary experts are expected to co-work in order to make this possible.

This study is not without limitations. One of the weaknesses is the number of subjects available, since we have to exclude a rather large number of subjects with incomplete records (23.7%) from the dataset. Such issue arises when dealing with archival data originating from routine clinical practice. Furthermore, the dataset is skewed towards negative cases which may have been the compounding factor in some of the classifier performance reported in this study. A more complete and balanced sample may give us better representation of the proposed model, thus warranting further investigation of the technique that will allow us to improve the current work. One possible solution to both problems, the skewed dataset and small sample size, is a synthetic data generator. As we are dealing with CT images, several studies have demonstrated the application of Generative Adversarial Network (GAN) to generate synthetic medical images through this generator-discriminator dual network [38–40]. This new data augmentation technique that works through image-to-image translation is a potential novel method on a limited dataset of medical images like ours. This could be a fascinating follow up work after this study, and in fact a preliminary screening of similar work has been started. Lastly, the present subjects included in this study originated from a single center. Additional studies in multiple centers are needed to confirm these results, particularly on the selection of the threshold value of dividing the solid versus non-solid dominant by the proposed biomarkers.

Despite these issues, we have confidence that it is possible to improve the current model such that it leads to more discoveries that can possibly enhance cancer care as more data become available. On top of this, with the recent pandemic of COVID-19, the proposed methodology holds potential in predicting the severity of COVID-19 in patients with lung cancer who tested positive with a SARS-CoV-2 by combining traditional biomarkers such as lab data including blood count, serum creatinine, and inflammatory markers with imaging data taken either on the same day, or the day after patient tested positive. Such a finding is an essential part of the international response to the pandemic, especially with regards to lung cancer patients.

## Conclusion

We have demonstrated that our customized model has shown better prognostic impact in comparison to the one-fits-all traditional model. The so-called patient specific model provides empirical evidence for the personalized medicine community as well as data-driven decision support system.

## Supplementary information

**Additional file 1 **: **Fig. 1.** The survival distribution is plotted as KM curves using each of the proposed biomarkers as a risk factor. The comparison groups are given as patients clustered into solid-dominant (SD) and non-solid dominant (NSD) tumor groups.

## Data Availability

The dataset was collected from TCIA (10.7937/K9/TCIA.2017.7hs46erv). This collection is freely available to browse, download, and use for commercial, scientific and educational purposes as outlined in the Creative Commons Attribution 3.0 Unported License. The dataset consists of DICOM images, AIM annotations, clinical data, and RNA sequence data (GSE103584).

## Abbreviations

IBMs: Imaging biomarkers
NSCLC: Non-Small Cell Lung Cancer
ROC: Receiver Operating Characteristic
AUC: Area under the ROC curves
KM: Kaplan-Meier
APACHE: acute physiology and chronic health evaluation
ICU: Intensive care unit
PaP: Palliative prognostic score
PPI: Palliative prognostic index
EMR: Electronic medical record
CBMs: Clinical biomarkers
IBMs: Imaging biomarkers
HBMs: Hybrid biomarkers
GGO: Ground Glass Opacity
TCIA: The cancer imaging archive
CT: Computed tomography
ROI: Region of interest
LR: Logistic Regression
RF: Random Forest
ANN: Artificial Neural Network
IDI: Integrated discrimination improvement
SD: Solid dominant
NSD: Non-solid dominant
