# Supplementary material for: Leveraging hybrid biomarkers in clinical endpoint prediction
Source: BMC Med Inform Decis Mak. 2020 Oct 7;20:255. doi: 10.1186/s12911-020-01262-3 (PMC7538849; doi:10.1186/s12911-020-01262-3)
Supplement: Supplementary file 1 — Additional file 1 : Fig. 1. The survival distribution is plotted as KM curves using each of the proposed biomarkers as a risk factor. The comparison groups are given as patients clustered into solid-dominant (SD) and non-solid dominant (NSD) tumor groups. [file 12911_2020_1262_MOESM1_ESM.docx]

**
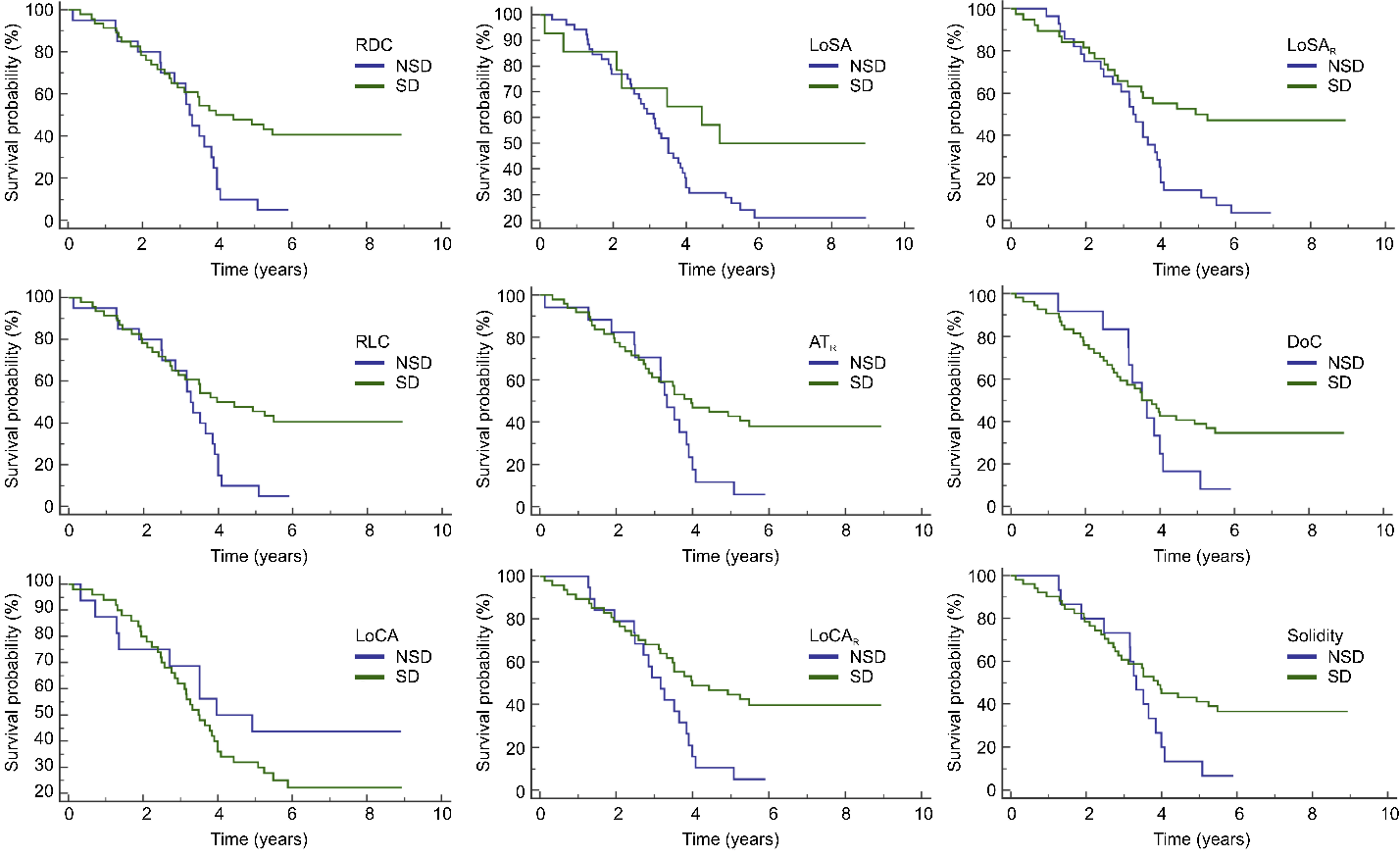
**

**Supplementary Fig 1.** The survival distribution is plotted as KM curves using each of the proposed biomarkers as a risk factor. The comparison groups are given as patients clustered into solid-dominant (SD) and non-solid dominant (NSD) tumor groups.
